# Semantic segmentation of autonomous driving scenes based on multi-scale adaptive attention mechanism

**DOI:** 10.3389/fnins.2023.1291674

**Published:** 2023-10-19

**Authors:** Danping Liu, Dong Zhang, Lei Wang, Jun Wang

**Affiliations:** ^1^School of Advanced Manufacturing Engineering, Hefei University, Hefei, China; ^2^State Key Laboratory of Automotive Simulation and Control, Jilin University, Changchun, China

**Keywords:** semantic segmentation, attention mechanism, autonomous driving, convolutional neural networks, deep learning

## Abstract

**Introduction:**

Semantic segmentation is a crucial visual representation learning task for autonomous driving systems, as it enables the perception of surrounding objects and road conditions to ensure safe and efficient navigation.

**Methods:**

In this paper, we present a novel semantic segmentation approach for autonomous driving scenes using a Multi-Scale Adaptive Mechanism (MSAAM). The proposed method addresses the challenges associated with complex driving environments, including large-scale variations, occlusions, and diverse object appearances. Our MSAAM integrates multiple scale features and adaptively selects the most relevant features for precise segmentation. We introduce a novel attention module that incorporates spatial, channel-wise and scale-wise attention mechanisms to effectively enhance the discriminative power of features.

**Results:**

The experimental results of the model on key objectives in the Cityscapes dataset are: ClassAvg:81.13, mIoU:71.46. The experimental results on comprehensive evaluation metrics are: AUROC:98.79, AP:68.46, FPR95:5.72. The experimental results in terms of computational cost are: GFLOPs:2117.01, Infer. Time (ms):61.06. All experimental results data are superior to the comparative method model.

**Discussion:**

The proposed method achieves superior performance compared to state-of-the-art techniques on several benchmark datasets demonstrating its efficacy in addressing the challenges of autonomous driving scene understanding.

## 1. Introduction

**Figure 1:**
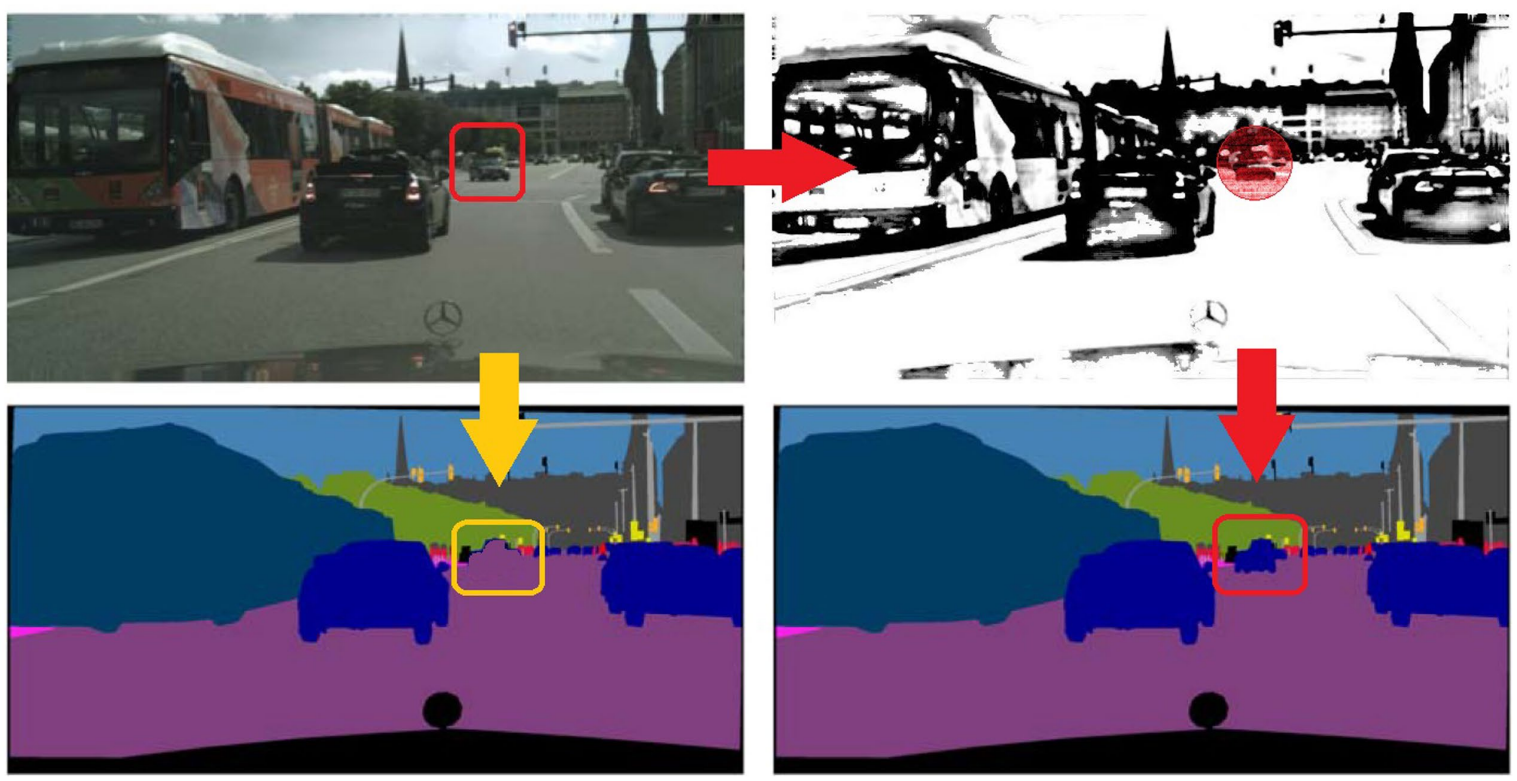
Examples of hazardous scenarios.

Over the past several decades, autonomous driving technology has made remarkable strides. The current bottleneck impeding its mass adoption is safety, as it directly pertains to human life and well-being. Autonomous vehicles are increasingly becoming integral across a multitude of scenarios—from daily living and work commutes to travel and leisure—where safety emerges as a critical factor governing their application. These self-driving platforms are fundamentally built upon sophisticated visual perception systems (Hubmann et al., 2018; Jin et al., 2021; Hu et al., 2023), in which semantic segmentation plays an essential role for pixel-level classification of camera images. While recent research has primarily focused on enhancing the accuracy of semantic segmentation, high-precision pixel-level classification of objects often relies on strong supervised learning methods trained on large, fully-annotated datasets. These models are consequently limited to classifying conventional objects—that is, categories predefined in the dataset—operating under the overly idealistic assumption that all objects in real-world driving environments remain constant. Unfortunately, the real world is ever-changing, and unpredictable situations can arise at any moment. For instance, an object with altered characteristics, such as an small obstacle in a driving scene in Figure 1, may not be properly identified by the model, which may overconfidently misclassify it into another category. Such scenarios pose serious safety risks and significantly hamper the practical deployment of deep learning algorithms in autonomous driving. Moreover, collecting a dataset that encompasses every conceivable variation is impractical. Driving environments that present significant challenges due to their dynamic nature fall under the category of hazardous scenarios, where all dynamic elements could be termed ‘anomalous obstacles.’ Therefore, it is crucial for a perception network to be trained to adapt to variations and anomalies in these risky settings.

Several studies have addressed the challenge of detecting variations and anomalous targets in hazardous driving scenarios (Lis et al., 2019; Doshi and Yilmaz, 2020; Xia et al., 2020; Blum et al., 2021; Vojir et al., 2021). One line of approaches employs uncertainty estimation techniques, intuitively based on the low prediction probabilities associated with anomalous targets. These methods design specific functions to compute uncertainty probabilities and subsequently generate anomaly scores. However, these techniques often yield noisy and imprecise detection results due to the model’s overconfidence in identifying anomalous targets. Another primary approach involves augmenting the training pipeline with additional tasks specifically for detecting anomalous obstacles. Some methods employ external out-of-distribution (OoD) datasets as training samples for this category, while others utilize feature reconstruction techniques to either manually design or learn the features of unknown classes to distinguish anomalies. Generative models are then used to resynthesize the input images. Although these methods have proven effective, they are either computationally expensive in terms of inference time or labor-intensive in their implementation. Moreover, the retraining process may compromise the original network’s performance in semantic segmentation. Therefore, there is a pressing need for more balanced solutions for perceiving and segmenting variations and anomalous objects in hazardous scenarios. The ideal approach should enhance the performance of uncertainty methods without significantly increasing computational overhead or training complexity, all while preserving the accuracy of semantic segmentation.

Human attention mechanisms serve as the foundation for various cognitive processes, allowing us to selectively focus on specific stimuli from an array of available inputs for deeper processing. While psychology offers critical methodologies for studying these attention mechanisms, neuroscience also stands as a primary field in which they are explored (Desimone and Duncan, 1995). Human attention can be conceptualized as a filtering process, determining which pieces of information merit further consideration and which should be disregarded (Treisman and Gelade, 1980). Psychological research delves into the behavioral aspects of attention, such as its selectivity, concentration, and shifting focus. Extensive inquiries into the operational aspects of attention have been made through experiments, observations, and surveys, covering theories of selective attention, filtering models, theories of attention allocation, and the attentional blink, among others (Broadbent, 1958; Kahneman, 1973; Raymond et al., 1992). Neuroscience examines the neural underpinnings of attention, identifying specific brain regions involved in the attention process. Utilizing functional Magnetic Resonance Imaging (fMRI) and electrophysiological techniques, scientists have identified the prefrontal and parietal cortices as key areas for regulating attention (Corbetta and Shulman, 2002), with additional research focusing on neurotransmitter systems and neural oscillations (Arnsten and Li, 2005; Jensen and Mazaheri, 2010). Given that attention mechanisms are integral to human cognition and crucial for learning, memory, decision-making, and other cognitive functions, they have inspired research and applications in computer science and artificial intelligence. In fields ranging from resource allocation to state-of-the-art deep learning models—particularly in scenarios dealing with big data and large volumes of information—attention mechanisms have found robust applications (Mnih et al., 2014; Bahdanau et al., 2015; Ma et al., 2019). Drawing inspiration from psychological and neuroscience research into attention mechanisms, significant progress has also been made in developing attention algorithms within the domain of artificial intelligence (Vaswani et al., 2017; Nobre and van Ede, 2018; Cichy and Kaiser, 2019).

Inspired by human attention mechanisms, humans demonstrate remarkable environmental perception skills, effortlessly identifying invariant and ordinary elements amidst variations and anomalies such as large-scale changes in object dimensions, occlusions, and diverse object appearances. This keen attention to the constant and ordinary amidst flux and irregularities equips humans with robust capabilities for environmental perception. How might this attention paradigm be mapped onto the domain of semantic segmentation in autonomous driving scenes? First, by analyzing and constructing the feature attributes associated with variations and anomalies in hazardous scenarios; and second, by aligning these identified feature attributes with the most fitting attention mechanisms.

One of the most pervasive attributes of variation and anomaly in autonomous driving scenarios is the substantial and high-frequency scale change of environmental objects. Objects may vary considerably in size and shape, and can be particularly challenging to recognize at differing image resolutions. For instance, a distant car may appear small in the image, whereas a nearby car would be considerably larger, leading to anomalies such as two objects at different distances with similar scales and contours being misperceived as the same category. To address this issue, we employ a scale attention mechanism that operates over multiple image scales within the network architecture. These results are then integrated to enhance the accuracy and robustness of semantic segmentation, thereby providing more reliable and granular information for autonomous driving scenarios.

Due to the spatially diverse distribution of objects at different scales—for instance, distant vehicles may occupy a diminutive spatial footprint, while nearby pedestrians may occupy a more substantial one—a scale attention mechanism necessitates integration with spatial attention. Without such a fusion, the model may struggle to ascertain the relative spatial positions and importance of differently sized structures or objects. For example, a distant small vehicle might be semantically more critical than a proximal large tree, but in the absence of spatial context, the model might disproportionately focus on the tree. Additionally, spatial attention allows the model to home in on partially obscured yet crucial areas, such as the legs or head of an obstructed pedestrian. Given that different features or attributes may reside in different channels—for instance, some channels may prioritize edge information, while others may focus on texture or color information—structures or objects of different scales may exhibit diverse feature expressions across these channels. For a scale attention mechanism to properly weight these features, channel attention integration becomes necessary, failing which could lead to information loss or confusion at certain scales. Moreover, objects in driving environments display various characteristics owing to changes in lighting, weather, and object types, among other factors. For instance, the same object category—such as a car—can display significant variations in color, model, and design. Since different appearance features may be distributed across different channels, channel attention allows the model to focus on key channels instrumental in identifying specific appearances.

This paper introduces a Multi-Scale Adaptive Attention Mechanism (MSAAM) for Semantic Segmentation in Autonomous Driving Scenes. Initially, a scale attention module is incorporated at the end of the Convolutional Neural Network (CNN) encoder. Subsequently, spatial and channel attention models are synergistically integrated to enhance the performance of the multi-scale attention mechanism. Building on this, a composite weighting model encompassing scale, spatial, and channel attention is established. This model is trained through a compact neural network to meet the requirements for adaptive weighting and employs the Softmax function to ensure the sum of the weights equals one, thereby preventing disproportionately large weights. Finally, an attention-specific loss function is proposed to further amplify the distance between the attention values focused on specific pixels and those on the remaining pixels. These methodologies allow us to train a semantic segmentation network based on MSAAM, effectively addressing the perceptual challenges posed by hazardous scenarios in autonomous driving, such as large-scale variations, occlusions, and diverse object appearances, among others.

The main contributions of our work are as follows:

This paper introduces the Multi-Scale Adaptive Attention Mechanism (MSAAM) specifically designed for semantic segmentation in driving scenarios. It is an attention mechanism that seamlessly integrates three channels—scale, spatial, and channel—and adaptively allocates their weights.

The multi-scale adaptive attention model that fuses multiple channels is adept at handling various attributes encountered in scenes, such as large-scale variations, occlusions, and diverse object appearances. Moreover, this attention model is highly modular and can be flexibly adapted to integrate with various Convolutional Neural Network (CNN) architectures, essentially offering a plug-and-play solution.

Our approach improves the performance of pixel-level semantic segmentation without substantially increasing the number of parameters or complicating the training process.

## 2. Related work

In the realm of hazardous scenario analysis, research work predominantly focuses on two main approaches for detecting variations and abnormal feature attributes: one that leverages uncertainty estimation and another that incorporates additional training tasks. This article also explores studies relevant to multi-scale attention mechanisms, which is the focus of our work. In this section, we provide an overview of research conducted in these three key areas.

### 2.1. Anomaly segmentation via uncertainty estimation

Methods based on uncertainty estimation serve as the most straightforward approach in abnormality detection, where uncertainty scores are utilized to identify obstacles on the road. Early studies employed Bayesian neural networks and Monte Carlo dropout to assess uncertainty. However, these techniques are often slow in inference and prone to boundary misclassifications (Kendall et al., 2015; Kendall and Gal, 2017; He et al., 2020). Alternative approaches focus on utilizing maximum softmax probabilities or maximum logits to improve uncertainty assessment, but these too suffer from the issue of boundary misclassification (Hendrycks and Gimpel, 2016; Jung et al., 2021; Hendrycks et al., 2022). Generally speaking, without additional fine-tuning using outlier data, methods based on uncertainty tend to perform poorly in terms of overconfidence and false positives at boundaries.

### 2.2. Anomaly segmentation via introducing additional training tasks

Another approach to abnormal segmentation involves incorporating extra training tasks. These tasks primarily fall under three categories: feature reconstruction, leveraging auxiliary datasets, and image re-synthesis. Feature reconstruction methods operate by analyzing the normality and deviations in the input features but are dependent on precise pixel-level segmentation (Creusot and Munawar, 2015; Di Biase et al., 2021). Methods based on auxiliary datasets employ external data to enhance detection accuracy but struggle to capture all potential anomalies, compromising the model’s generalizability (Bevandic´ et al., 2019; Chan et al., 2021). Image re-synthesis techniques, such as those employing autoencoders and Generative Adversarial Networks (GANs), create more diverse abnormal samples but at the cost of computational complexity and extended inference time (Ohgushi et al., 2020; Tian et al., 2021). While these additional training tasks contribute to improving abnormality detection, they may also adversely impact the primary task, i.e., semantic segmentation performance.

### 2.3. Multi-scale attention mechanisms for image segmentation or fine-grained image classification

Effective learning of multi-scale attention regions is pivotal in the domains of image segmentation and fine-grained image classification (Ge et al., 2019; Zheng et al., 2019). Earlier research largely relied on manually annotated object bounding boxes, a process that is both time-consuming and impractical. Xiao et al. were the first to introduce a multi-scale attention model that does not depend on manual annotation, incorporating both object-level and part-level attention (Xiao et al., 2015). More recent studies have evolved to be more intricate, involving adaptive region localization, weakly-supervised learning, and Feature Pyramid Networks (Fu et al., 2017; Rao et al., 2019; Ding et al., 2021). These advancements contribute to more precise localization and classification of target areas, thereby enhancing the performance of pixel-level segmentation or fine-grained classification (Li et al., 2016a,b; Nian et al., 2016; Zhang et al., 2019, 2020; Jiang et al., 2020; Liu et al., 2021).

## 3. Methodology

This section elucidates the Multi-Scale Adaptive Attention Mechanism (MSAAM) approach that we employ for semantic segmentation in autonomous driving scenes. Initially, in Subsection 3.1, we articulate the motivations underlying our methodology. Following this, Subsection 3.2 presents an overview of the comprehensive architecture of MSAAM. Subsection 3.3 details the multi-scale attention module, while Subsection 3.4 describes a weight-adaptive fusion attention system.

### 3.1. Motivation

Human attention mechanisms assist us in selecting and focusing on a particular stimulus among various inputs for in-depth processing. This mechanism is not only a focal point in psychological research but also a principal area of study in neuroscience. Psychology investigates the behavioral characteristics of attention, utilizing a range of experiments and questionnaires to understand how attention is selected and allocated. Neuroscience, on the other hand, delves into the brain regions responsible for attention, employing technologies such as fMRI and electrophysiology. Attention plays a crucial role in cognitive functions like learning, memory, and decision-making. Inspired by these insights, the fields of computer science and artificial intelligence have also begun to explore and implement attention mechanisms, especially in contexts that involve large-scale data and high information volume. Advances in attention mechanisms within artificial intelligence have been made by drawing upon foundational research in psychology and neuroscience.

Inspired by human attention mechanisms, we can identify stability and regularity amidst environmental variations and anomalies, thereby perceiving the environment more effectively. How can such an attention paradigm be applied to semantic segmentation in autonomous driving scenarios? First, it involves analyzing and identifying the characteristics of variations and anomalies in hazardous scenes; second, it calls for choosing suitable attention mechanisms tailored for these specific traits.

In autonomous driving scenes, rapid and substantial changes in object scale pose a significant challenge. For instance, cars at varying distances appear drastically different in size within the same image, potentially leading to erroneous identification. To tackle this issue, we employ scale attention mechanisms to process multiple image scales and integrate the results. This enhances the accuracy and robustness of semantic segmentation, making autonomous driving more reliable.

In autonomous driving contexts, both the scale and spatial positioning of objects are of paramount importance. For example, a distant car may hold more significance than a nearby tree, yet the model may overemphasize the tree due to a lack of spatial context. Therefore, scale attention must be combined with spatial attention to comprehend the relative positioning and importance of objects in space. Spatial attention also helps the model focus on partially occluded yet crucial areas. Additionally, object features of different scales and appearances might reside in different channels, such as edge or color information. To avoid losing or confusing these details, the scale attention model also incorporates channel attention. In this way, the model can more accurately identify a variety of appearances under different lighting conditions, weather, and object types.

### 3.2. Overall architecture

Semantic segmentation models are generally formulated as encoder-decoder architectures. An input image is initially transformed into high-dimensional features via the encoder. Subsequently, with these intermediate features as input, the MSAAM first infers a two-dimensional attention map. Importantly, attention should not be unbounded. A constant-sum constraint on attention values forces pixels within the attention map to compete against each other for maximal gain, thereby circumventing the pitfall of the model setting all attention values unfavorably high. We then select multi-layer, multi-scale features generated by the encoder and fuse them with the attention map. These fused features are fed into the decoder network to produce the predictive output. To widen the gap in attention values between focus pixels and other pixels, we introduce a penalty term in the loss function, termed as MSAAM Loss. Finally, the network’s predictive output is combined with MSAAM’s attention map to generate the ultimate integrated prediction.

**Figure 2:**
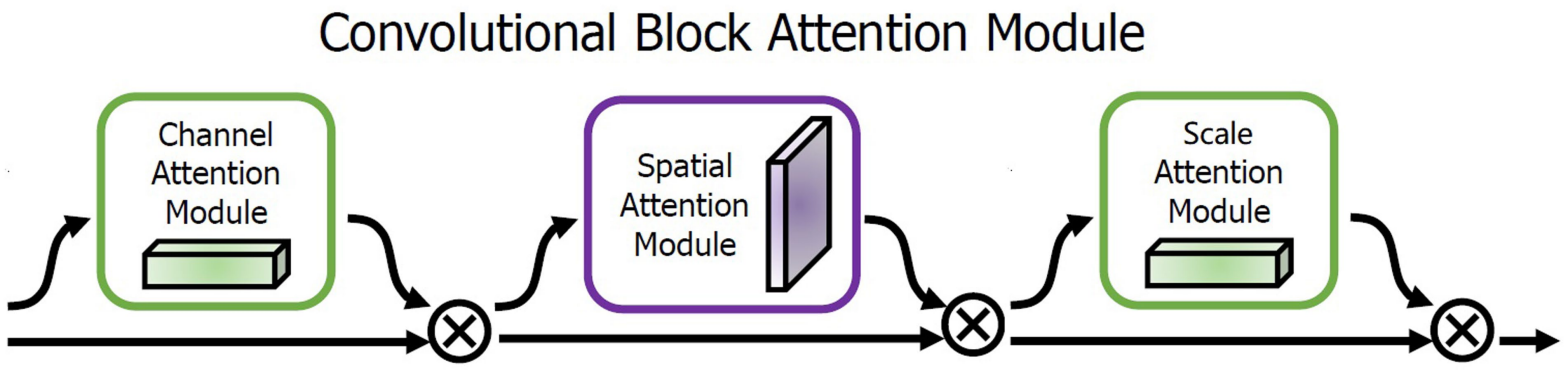
Architecture of the MSAAM attention mechanism.

Within the architecture, the MSAAM module situated between the encoder and the decoder serves as the linchpin for the attention mechanism. Initially, a Pyramid Attention Module is integrated at the terminal phase of the encoder. This module employs Pyramid Pooling to capture information across different scales, thereby establishing a multi-scale attention mechanism. Subsequently, we utilize the Convolutional Block Attention Module (CBAM) to concurrently address both spatial and channel attention. CBAM enriches contextual information by employing Global Average Pooling and Global Max Pooling techniques. To precisely calculate the weights across the three dimensions—scale, space, and channel—we have engineered a miniature neural network. This network comprises several fully connected layers and a Softmax layer, designed to learn the aggregate attention weights across different dimensions. As a specific implementation detail, Gated Recurrent Units (GRU) are employed to update the weights for each dimension, thus constructing a weight-adaptive model. The basic architecture of attention is shown in Figure 2.

### 3.3. Multi-scale attention module

Addressing the large-scale variations of objects poses a significant challenge for semantic segmentation in autonomous driving scenarios. Integrating a multi-scale attention mechanism into the segmentation process ameliorates these challenges by enabling the model to focus on regions of varying sizes.

The Pyramid Pooling Attention module (PSA) is specifically designed to capture contextual information across different dimensions and spatial resolutions. Traditional attention mechanisms often operate at a single scale, which could limit their ability to understand either broader or more nuanced details. In contrast, pyramid models, by creating representations at various granularities, can effectively tackle the multi-scale challenges inherent in computer vision. These representations offer a more comprehensive understanding of the scene, which is crucial for enhancing segmentation performance in diverse and dynamically changing environments, such as those encountered in autonomous driving.

The scale-wise attention module $f^{sc}$ in our framework is a sophisticated operation that effectively combines the input feature map $F_{\mathrm{in}}$ with an attention map produced by the PSA module. Mathematically, it is represented as:


(1) $$f^{sc}\left(F_{\mathrm{in}}\right)=F_{\mathrm{in}}+F_{\mathrm{in}}⊙PSA\left(F_{\mathrm{in}}\right)$$

in this context, symbolizes the scale-wise attention module, $F_{\mathrm{in}}$ is the input feature map, ⊙ stands for element-wise multiplication, and PSA denotes the Pyramid Pooling Attention module. The essence of this formula is that given an intermediate feature map, our module produces an attention map through the Pyramid Pooling Attention module and then multiplies this attention map with the input feature map, achieving adaptive feature refinement.

The definition of the Pyramid Pooling Attention module PSA is as follows:


(2) $$PSA\left(F_{\mathrm{in}}\right)=\mathrm{softmax}\left(\overset{N}{\underset{i=1}{\sum }}\omega_{i}P_{i}*F_{\mathrm{in}}\right)$$

in this equation, N represents the number of layers in the pyramid, $P_{i}$ refers to the pooling operation at the *i*-th layer, $w_{i}$ is the weight for that layer, and ∗ denotes the convolution operation. The resulting attention map amalgamates information from different scales by using a weighted combination of pyramid layers.

### 3.4. Weight-adaptive fusion attention system

Following the scale attention layer, we integrate both spatial and channel attention layers, formalized as follows:


(3) $$f^{sp}\left(F\right)=\sigma \left(\mathrm{Con}v_{7\times 7}\left(\frac{1}{C\left(F\right)}\underset{\forall j}{\sum }f\left(F_{i},F_{j}\right)g\left(F_{j}\right)\right)\right)$$

where, $f^{sp}$ represents the spatial attention module, σ denotes the sigmoid function, ${\mathrm{Conv}}_{7\times 7}$ stands for a convolutional layer with a kernel size of 7×7 , $F_{i}$ and $F_{j}$ represent the input features from any two positions, *f* is a function for calculating the relationship between two positions, *g* is a function to compute the embedding of input features, and *C* signifies a normalization factor.


(4) $$f^{c}\left(F_{\mathrm{in}}\right)=F_{\mathrm{in}}⊙\sigma \left(W_{3}\left(\delta \left(W_{2}\delta \left(W_{1}F_{\mathrm{in}}\right)\right)+b_{3}\right)\right)$$

here, $f_{c}$ indicates the channel-wise attention module, $F_{\mathrm{in}}$ is the input feature map, ⊙ refers to element-wise multiplication, σ represents the Sigmoid function, δ is the ReLU function, $W_{1}$ , $W_{2}$ , and $W_{3}$ are convolution kernel parameters, and $b_{3}$ is the bias parameter.

To accurately calculate the weights across three dimensions—scale, space, and channel—a compact neural network is designed. It consists of several fully connected layers and a Softmax layer, employed for learning the composite attention weights across different dimensions. Specifically, Gated Recurrent Units (GRU) are utilized to update the weights for each dimension. The formal definition is:


(5) $$h_{t}=GRU\left(\begin{array}{l} W_{sc}\cdot f^{sc}\left(F_{\mathrm{in}}\right)+W_{sp}\cdot f^{sp}\left(F\right)\\ +W_{c}\cdot f^{c}\left(F_{\mathrm{in}}\right),\;h_{t-1} \end{array}\right)$$

here, $h_{t}$ represents the hidden state at time *t*, employed for weight calculation. $W_{sc}$ , $W_{sp}$ , and $W_{c}$ are weight matrices corresponding to scale, space, and channel, respectively.

The computation of the weights can be realized through a straightforward fully connected layer:


(6) $$\alpha_{sc},\alpha_{sp},\alpha_{c}=\mathrm{Softmax}\left(W_{h}\cdot h_{t}\right)$$

here, $\alpha_{sc},\alpha_{sp},\mathrm{and}\;\alpha_{c}$ denote the weights across the three dimensions.

To enlarge the attention-value gap between the focus pixels and the remaining pixels, a penalty term is introduced in the loss function, known as MSAAM Loss, defined as:


(7) $$\begin{array}{l} \mathrm{MSAAMLoss}=\mathrm{CrossEntropy}\left(Y,\overset{\wedge }{Y}\right)+\\ \;\lambda \left(Var\left(\alpha_{sc}\right)+Var\left(\alpha_{sp}\right)+Var\left(\alpha_{c}\right)\right) \end{array}$$

here, *Y* is the ground truth, $\overset{\wedge }{Y}$ is the model prediction, and λ is a hyperparameter that balances the importance of the two terms. $Var\left(\alpha \right)$ indicates the variance of the weights; a higher variance implies that the model has allocated significantly different weights across different scales, spaces, or channels—something we wish to encourage.

In summary, the GRU model maintains a hidden state that captures the significance of the scale, space, and channel information observed thus far. These weights are normalized through a Softmax layer for subsequent use in the attention mechanism. The MSAAM Loss is an extension of the basic cross-entropy loss for semantic segmentation tasks. The second term is a variance term, intended to encourage the model to allocate different weights across the three disparate dimensions—scale, space, and channel—to enhance the model’s diversity and robustness. Finally, we merge the network’s predicted output with the MSAAM attention map to obtain the final integrated prediction. Such a design helps the model better capture the importance across different scales, spaces, and channels, while also encouraging greater attention to the variances among these dimensions.

## 4. Experiments

### 4.1. Datasets

MSAAM is proposed to improve the semantic segmentation for autonomous driving cars in street scenes, we empirically verify it on CamVid dataset and Cityscapes dataset in this section. CamVid contains 367 training images, 101 validation images, and 233 test images. The resolution of images in this dataset is 960 × 720 which will be downsampled to 480 × 360 for accelerating the training stage of SS models. Cityscapes is comprised of a large, diverse set of high-resolution (2048 × 1,024) images recorded in streets, where 5,000 of these images have high quality pixel-level labels of 19 classes and results 9.43 × 10^9 labeled pixels in total. Following the standard setting of Cityscapes, the 5,000 images are split into 2,975 training and 500 validation images with publicly available annotation, as well as 1,525 test images with annotations withheld and comparison to other methods is performed via a dedicated evaluation server.

### 4.2. Experimental setup

#### 4.2.1. Implementation details

We adopt DeepLabv3+ with ResNet101 backbone for our segmentation architecture with the output stride set to 8. MSAAM is incorporated at the end of the encoder. We train our segmentation networks on Cityscapes. We use the same pre-trained network for all experiments.

To avoid over-fitting, common data augmentations are used as preprocessing, including random flipping horizontally, random scaling in the range of [0.5, 2], random brightness jittering within the range of [−10, 10], and random crop of 512 × 512 image patches. For training, we use the Adam optimizer (Kahneman, 1973) with an initial learning rate of 0.0003 and weight decay of 0.00001. The learning rate is scheduled by multiplying the initial learning rate with ${\left(1-\frac{\mathrm{epoch}}{\mathrm{maxEpoches}}\right)}^{0.9}$ . All models are trained for 80 epochs with minibatch size of 8.

#### 4.2.2. Evaluation metrics

For quantitative evaluation, mean of class-wise Intersection over Union (mIoU) are used. We also use the class accuracy (ClassAcc) to evaluate the performance of compared methods on different datasets. We compare the performance by the area under receiver operating characteristics (AUROC) and average precision (AP). In addition, we measure the false positive rate at a true positive rate of 95% (FPR95) since the rate of false positives in high-recall areas is crucial for safety-critical applications.

#### 4.2.3. Baselines

In Cityscapes dataset, we pick up 19 the most frequently occurred classes from the original 35 classes based on the official evaluation metrics (Raymond et al., 1992), and their importance groupings from trivial to important are.

Group 1 = {Sky, Building, Vegetation, Terrain, Wall};

Group 2 = {Pole, Road, Sidewalk, Fence};

Group 3 = {Traffic sign, Traffic light, Car, Truck, Bus, Train, Motorcycle, Person, Rider, Bicycle};

We compare our method with important approaches including Synboost, SML, Max logits, Entropy, MSP, Energy, SynthCP, Meta-OoD (Broadbent, 1958; Treisman and Gelade, 1980; Desimone and Duncan, 1995; Hubmann et al., 2018; Lis et al., 2019; Doshi and Yilmaz, 2020; Xia et al., 2020; Blum et al., 2021; Vojir et al., 2021) on test sets of CamVid and on validation sets of Cityscapes. Note that Synboost and SynthCP requires additional training of extra network and utilizing OoD data. Energy and Meta-OoD requires additional training of extra component or network. SML, Max logits, Entropy and MSP do not require additional training or utilize external datasets.

### 4.3. Evaluation results

In this section, we compare the performances of important approaches with MSAAM under the above experimental settings. The experimental results of compared methods on the investigated classes of the two datasets are shown in Tables 1–4, respectively. A more comprehensive set of quantitative analysis metrics is shown in Table 5.

**Table 1:** The comparison results (%) of various methods on the Groups 1 and 2 of Camvid Dataset.

Models	Group 1			Group 2			
	Sky	Building	Tree	Column	Road	Sidewalk	Fence
Synboost	97.06	71.61	77.84	34.31	93.41	90.35	53.57
SML	93.77	86.75	83.29	21.59	98.28	86.38	31.38
Max logits	94.21	71.6	**90.88**	48.92	93.17	88.78	45.19
Entropy	89.98	**88.92**	84.58	9.71	94.56	81.27	19.86
MSP	93.38	87.45	83.87	17.23	90.24	88.76	43.33
Energy	85.12	86.4	71.77	20.23	**98.66**	75.03	25.56
SynthCP	94.44	78.71	88.09	42.28	98.29	94.57	44.84
Meta-OoD	**97.87**	86.28	81.18	30.04	98.66	86.04	32.74
MSAAM	96.82	75.16	82.81	**60.36**	92.11	**95.19**	**62.02**

The bold values mean highlighting the best results in the data comparison.

**Table 2:** The comparison results (%) of various methods on the Group 3 of Camvid Dataset.

Models	Group 3					
	Sign	Car	Pedestrian	Bicyclist	ClassAvg	mIoU
Synboost	50.49	82.92	67.21	33.11	71.21	51.19
SML	40.79	80.28	59.93	15.19	64.21	51.08
Max logits	26.58	79.38	39.43	42.29	67.88	52.34
Entropy	0.72	75.37	25.09	0.48	52.32	45.35
MSP	32.33	83.53	36.08	23.45	58.91	47.71
Energy	29.39	80.82	48.08	28.25	60.11	48.51
SynthCP	43.37	76.01	66.39	52.05	72.51	55.31
Meta-OoD	19.58	76.56	37.65	36.08	63.07	53.21
MSAAM	**67.57**	**91.63**	**78.17**	**62.51**	**74.81**	**55.87**

The bold values mean highlighting the best results in the data comparison.

**Table 3:** The comparison results (%) of various methods on the Groups 1 and 2 of Cityscapes Dataset.

Models	Group 1					Group 2			
	Sky	Building	Vegetation	Terrain	Wall	Pole	Road	Sidewalk	Fence
Synboost	95.57	94.27	94.73	**77.53**	57.85	**74.28**	94.89	84.84	64.16
SML	99.21	92.21	**97.64**	66.35	35.54	49.66	98.36	82.78	59.97
Max logits	98.58	85.37	95.73	52.48	43.38	59.65	93.39	86.97	36.92
Entropy	94.17	92.95	93.06	61.71	12.45	40.11	96.48	81.23	43.69
MSP	92.19	81.63	93.44	64.77	32.95	30.43	97.81	80.23	35.33
Energy	93.56	**95.02**	90.73	41.24	16.85	28.79	98.61	77.03	25.84
SynthCP	**99.52**	90.52	90.79	76.21	**68.52**	70.03	96.80	87.28	**64.95**
Meta-OoD	94.67	93.04	93.72	75.85	58.48	67.48	**99.62**	92.61	59.81
MSAAM	93.55	86.86	91.26	67.14	54.47	70.73	94.66	**94.48**	62.03

The bold values mean highlighting the best results in the data comparison.

**Table 4:** The comparison results (%) of various methods on the Group 3 of Cityscapes Dataset.

Models	Group 3											
	Traffic Sign	Traffic Light	Car	Truck	Bus	Train	Motorcycle	Person	Rider	Bicycle	ClassAvg	mIoU
Synboost	75.96	71.18	98.92	68.10	73.87	61.07	42.50	87.29	57.79	81.82	74.66	58.20
SML	62.75	27.42	91.60	0.00	62.93	0.00	0.00	83.05	0.00	63.91	58.52	44.84
Max logits	55.08	21.27	96.42	44.86	41.29	16.94	3.14	67.28	39.47	66.89	59.72	42.58
Entropy	15.03	7.57	90.01	13.20	1.04	52.52	2.55	62.68	0.00	50.58	45.80	38.92
MSP	45.98	14.01	91.50	1.34	29.85	1.02	0.52	67.59	3.57	61.25	48.52	40.20
Energy	42.59	11.60	93.85	2.25	3.51	11.83	0.29	61.65	0.10	57.02	46.28	37.76
SynthCP	83.64	77.40	95.90	77.59	87.49	78.30	56.92	85.37	66.96	85.38	75.69	67.89
Meta-OoD	74.72	67.08	96.56	72.26	82.57	72.02	53.00	87.59	64.57	81.22	79.99	69.34
MSAAM	**89.55**	**81.61**	**99.36**	**88.85**	**89.52**	**85.82**	**57.41**	**89.11**	**70.11**	**89.64**	**81.13**	**71.46**

The bold values mean highlighting the best results in the data comparison.

**Table 5:** The comparison results of various methods on AUROC, AP, and FPR_95_.

Models	AUROC↑	AP↑	FPR95↓
Synboost	92.48	47.88	49.04
SML	96.77	50.09	17.37
Max logits	93.75	28.07	29.86
Entropy	90.39	21.93	34.75
MSP	88.26	14.85	33.97
Energy	92.61	30.30	38.37
SynthCP	89.34	22.26	32.72
Meta-OoD	97.38	67.41	13.76
MSAAM	**98.79**	**68.46**	**5.72**

The bold values mean highlighting the best results in the data comparison.

From the results shown in Tables 1, 2, we find that by embedding our MSAAM to the adopted deep models, the performance of the investigated important classes like sign/symbol, pedestrian, and bicyclist can be significantly improved when compared with the results of other approaches. Not surprisingly, the performance on unimportant classes such as sky, building, and tree weakly drop because they are the target of the attention mechanism. The performance gain of MSAAM over the second approach are 17.08, 8.1, 11.04, 10.46 on sign, car, pedestrian, bicyclist, respectively. Meanwhile, MSAAM achieve better performance than other approaches of ClassAvg and mIoU values.

From the results in Tables 3, 4, we observe that the important classes in Group 3 are segmented with very high performance by MSAAM. The performance gain of MSAAM on ClassAvg and mIoU are 1.14 and 2.12. For some unimportant classes in Group 1 and 2, the performances of the MSAAM-based model are inferior to the other models. However, they will not have a large impact on safe-driving as explained above.

To further evaluate the experimental results through quantitative analysis, we conducted a data analysis on the three metrics, AUROC, AP, FPR_95_ presented in Table 5. From the results, we observe that embedding our MSAAM to the adopted deep models, the performance achieved the best results compared to all other models. The performance gain of MSAAM on AUROC, AP and mIoU are 1.41, 1.05, 8.04, respectively.

### 4.4. Auxiliary hierarchical representation

To qualitatively analyze the experimental results, we design an algorithm to extract the weights from multiple attention modules. It then simplifies the attention pixels into rectangular blocks for visualization. This algorithm is named auxiliary hierarchical representation.

The original image dimensions are H×W×C . The attention weights $\alpha_{sc}$ , $\alpha_{sp}$ and $\alpha_{c}$ are extracted from the GRU model. In the Scale Attention Auxiliary Hierarchical Representation, the weight $\alpha_{sc}$ and the scale attention output $f^{sc}\left(F_{\mathrm{in}}\right)$ are utilized to compute an H×W scale weight matrix. In this matrix, the weight of each pixel (*i*, *j*) is the weighted sum of $\alpha_{sc}\cdot f_{ij}^{sc}$ across all scales, defined as follows:


(8) $${\mathrm{ScAH}}_{ij}=\underset{s}{\sum }\alpha_{sc,s}\cdot f_{s,ij}^{sc}$$

here, ScAH stands for Scale Attention Highlight.

In the case of Spatial Attention Auxiliary Hierarchical Representation, the weight $\alpha_{sp}$ and the spatial attention output $f^{sp}\left(F\right)$ are employed to calculate an H×W spatial weight matrix, defined as:


(9) $${\mathrm{SpAH}}_{ij}=\alpha_{sp}\cdot f_{ij}^{sp}$$

here, SpAH stands for Spatial Attention Highlight.

For Channel Attention Auxiliary Hierarchical Representation, the weight $\alpha_{c}$ and the channel attention output $f^{c}\left(F_{\mathrm{in}}\right)$ are used to compute an H×W channel weight matrix. Here, the weight of each pixel (*i*, *j*) is the weighted sum of $\alpha_{c}\cdot f_{ij}^{c}$ across all channels, defined as follows:


(10) $${CAH}_{ij}=\underset{c}{\sum }\alpha_{c,c}\cdot f_{c,ij}^{c}$$

in this context, CAH represents Channel Attention Highlight.

Upon the completion of the hierarchical model construction, the model undergoes normalization and color mapping to facilitate the high-contrast highlighting of attention regions. For an optimized visual experience, a simplified treatment is generally applied to the regions of attention.

After auxiliary hierarchical modeling is accomplished for all three attention mechanisms—scale, spatial, and channel—their respective weights are combined to create a rectangular attention visualization model, providing a more straightforward and interactive way to represent attention intervals.

Initially, the weights are amalgamated by integrating the weight matrices of Scale, Spatial, and Channel into a new weight matrix termed as Combined Attention Highlight, abbreviated as CoAH. The combination is formalized as:


(11) $${\mathrm{CoAH}}_{ij}=\alpha_{sc}\cdot ScAH_{ij}+\alpha_{sp}\cdot \mathrm{SpA}H_{ij}+\alpha_{c}\cdot CAH_{ij}$$

here, $\alpha_{sc}$ , $\alpha_{sp}$ , and $\alpha_{c}$ are normalized weights retrieved from the GRU model.

Subsequently, a simplified rectangular model is established. A simplification algorithm, such as a greedy algorithm or another optimization technique, is employed to identify a rectangular region with the highest average attention weight. Assuming the rectangular region is defined by the top-left corner $\left(x_{1},y_{1}\right)$ and the bottom-right corner $\left(x_{2},y_{2}\right)$ , the average weight for this area is computed as follows:


(12) $$\mathrm{AW}=\frac{1}{\left(x_{2}-x_{1}+1\right)\times \left(y_{2}-y_{1}+1\right)}\overset{x_{2}}{\underset{i=x_{1}}{\sum }}\overset{y_{2}}{\underset{j=y_{1}}{\sum }}\;CoAH_{ij}$$

in this equation, AW stands for Average Weight.

**Figure 3:**
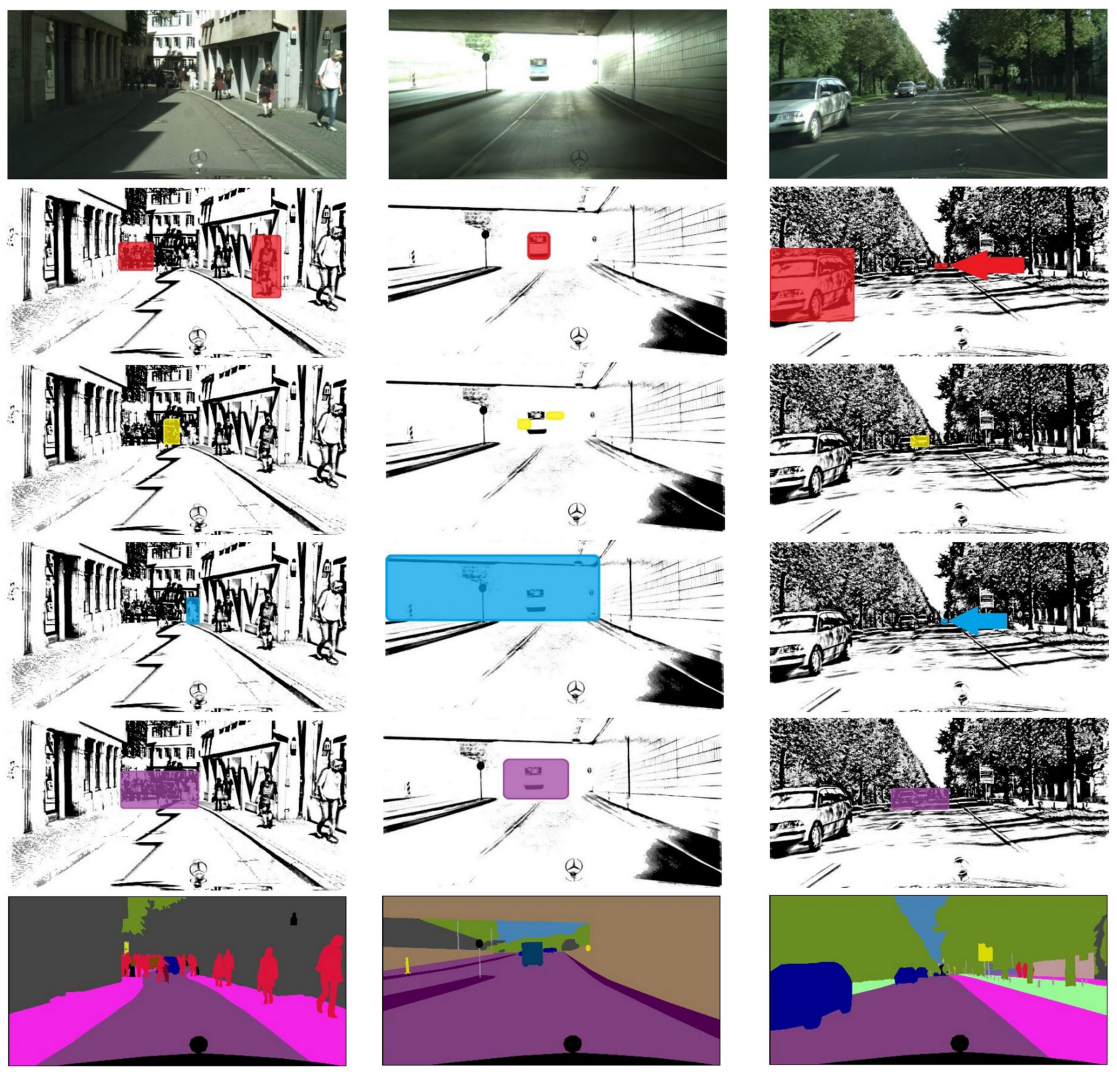
Visualization of the working process of the MSAAM attention mechanism.

The visualization of the auxiliary hierarchical representation based on the MSAAM attention mechanism is shown in Figure 3. Scale attention captures objects of the focused category at different sizes. Subsequently, spatial attention tends to prioritize obscured targets, while channel attention is inclined toward targets with significant appearance variations. Both spatial and channel attentions assist scale attention in optimizing the areas and objects of focus, culminating in an integrated attention model. Auxiliary hierarchical representation is for the purpose of visualizing this process.

### 4.5. Ablation study

We integrated the MSAAM into the models that do not require additional training or utilize external datasets. These models include SML, Max logits, Entropy and MSP. From the results in Table 6, we observe that all performance metrics of every model improved. The experimental outcomes underscore the versatility and effectiveness of MSAAM.

**Table 6:** Comparison of metric gains after embedding our MSAAM to models that do not require additional training or utilize external datasets.

	AUROC↑	AP↑	FPR95↓	mIoU
SML + MSAAM	+0.63	+1.66	+2.70	+1.49
Max logits + MSAAM	+0.10	+6.90	+2.47	+0.42
Entropy + MSAAM	+1.54	+7.21	+1.85	+1.65
MSP + MSAAM	+1.45	+2.34	+1.63	+0.19

### 4.6. Comparison on effectiveness

**Figure 4:**
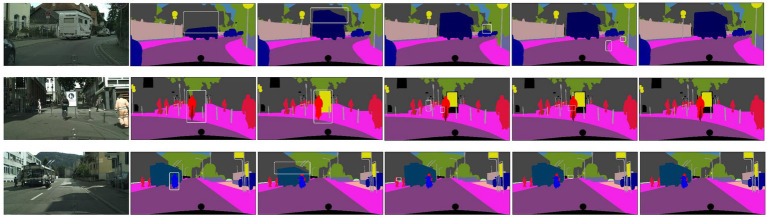
Comparison on effectiveness.

To demonstrate the effectiveness of MSAAM on Cityscapes dataset, Figure 4 shows some representative segmentation results of the SML, Max logits, Entropy and MSAAM. We find that the interested regions segmented by the MSAAM are highly compact, and the shapes of the segmented objects are also more close to that of the ground truth. Therefore, MSAAM is effective in emphasizing the small but critical targets, and thus is useful for semantic segmentation tasks.

### 4.7. Comparison on computational cost

To demonstrate that our method requires a negligible amount of computation cost, we report GFLOPs (i.e., the number of floating-point operations used for computation) and the inference time. As shown in Table 7, our method requires only a minimal amount of computation cost regarding both GFLOPs and the inference time compared to the other approaches.

**Table 7:** Comparison on computational cost.

Models	GFLOPs	Infer. Time (ms)
Synboost	4762.15	165.27
SML	2139.86	61.41
Max logits	2169.32	66.45
Entropy	2631.33	72.88
MSP	2431.59	77.12
Energy	2201.09	70.15
SynthCP	4551.11	146.91
Meta-OoD	4776.81	150.84
MSAAM	2117.01	61.06

## 5. Conclusion

In this paper, we present the Multi-Scale Adaptive Attention Mechanism (MSAAM), a specialized framework tailored for enhancing semantic segmentation in automotive environments. The attention mechanism uniquely harmonizes three critical dimensions—scale, spatial context, and channel features—while adaptively balancing their respective contributions. By integrating these multi-faceted channels, MSAAM excels in addressing complex scene attributes such as scale discrepancies, object occlusions, and diverse visual appearances. Notably, the architecture of this attention mechanism is highly modular, enabling seamless incorporation into a wide array of Convolutional Neural Network (CNN) models. As a result, it serves as a versatile, plug-and-play component that augments pixel-level semantic segmentation performance without significantly inflating the parameter count or complicating the training regimen.

## Data availability statement

Publicly available datasets were analyzed in this study. This data can be found at: https://www.cityscapes-dataset.com/.

## Author contributions

DL: Writing – original draft. DZ: Writing – review & editing. LW: Writing – review & editing. JW: Writing – review & editing.
